# Relationship between rumen ciliate protozoa and biohydrogenation fatty acid profile in rumen and meat of lambs

**DOI:** 10.1371/journal.pone.0221996

**Published:** 2019-09-06

**Authors:** Alexandra Eduarda Francisco, José Manuel Santos-Silva, Ana Paula V. Portugal, Susana Paula Alves, Rui José B. Bessa

**Affiliations:** 1 Unidade Estratégica de Investigação e Serviços em Produção e Saúde Animal, Instituto Nacional de Investigação Agrária e Veterinária, Vale de Santarém, Portugal; 2 Centro de Investigação Interdisciplinar em Sanidade Animal, Avenida da Universidade Técnica, Lisboa, Portugal; 3 Faculdade de Medicina Veterinária, Universidade de Lisboa, Pólo Universitário do Alto da Ajuda, Lisboa, Portugal; University of Agriculture in Krakow, POLAND

## Abstract

This study investigated the associations between abundance of rumen ciliate protozoa and the proportion of the main bioactive fatty acids related to rumen biohydrogenation, as 18:0, *t*10-18:1, *t*11-18:1, *c*9,*t*11-18:2, 18:3n-3 and 18:2 n-6, in rumen and meat of growing lambs, using data derived from 3 production experiments. A global correlation analysis and a linear regression analysis considering the effect of the experiment were performed. Ten of the 86 lambs involved in the experiments did not present ciliate cells in rumen liquor and the remaining lambs presented an average of 1.35 × 10^6^ciliates / ml rumen liquor. From the nine genera of ciliates identified, *Entodinium* was the most abundant, averaging 1.17 × 10^6^ cells / ml of rumen liquor. A large variation among lambs was observed for both rumen concentration and community structure of ciliates. Rumen *t*11-18:1 (*P* < 0.001) and meat deposition of *t*11-18:1 (*P* < 0.001) and of *c*9,*t*11-18:2 (*P* < 0.001) increased linearly with total ciliates, whereas the *t*10/*t*11 ratio in rumen (*P* = 0.002) and in meat (*P* = 0.036) decreased linearly. Entodiniomorphids seems to be strongly related with meat deposition of*t*11-18:1 and *c*9,*t*11-18:2 and with the reduction of the *trans*-10 shifted pathway. Completeness of RBH decreased linearly with Holotrichs (*P* = 0.029), Entodiniomorphids (*P* = 0.029), *Isotricha* (*P* = 0.011) and *Epidinium* (*P* = 0.027) abundances. Rumen 18:0 also decreased linearly with increasing counts of total ciliates (*P* = 0.015), Holotrichs (*P* = 0.020), Entodiniomorphids (*P* = 0.010) and *Isotricha* (*P* = 0.014). Rumen protozoa were positively linked with the deposition of healthy bioactive FA and simultaneously negatively associated with the occurrence of *trans*-10 shift.

## Introduction

By rumen biohydrogenation (RBH), the dietary unsaturated fatty acids (UFA) are converted by rumen microbiota to saturated fatty acids (SFA) which negatively impact to human health. However, RBH is not complete and a variable proportion of UFA submitted to RBH end up as a isomerized or partially hydrogenated FA, hereafter named biohydrogenation intermediate (BI), that are absorbed and incorporated in ruminant´s milk and meat fat[1]. Some of them, present healthy properties such as vaccenic (*t*11-18:1) and rumenic (*c*9,*t*11-18:2) acids. The fibrolytic bacteria, which are believed to be the main responsible for RBH pathways that generate *t*11-18:1 and *c*9,*t*11-18: 2 as the main octadecaenoic and octadecadienoic BI[2], are very sensitive to low ruminal pH[3]. Diets rich in quickly fermentable carbohydrates, like starch, led to low rumen pH, impacting the ecosystem equilibria and the balance between microbial populations. Such conditions favour the amilolytic populations associated to the occurrence of a shift in RBH pathways where the major BI becomes *t*10-18:1 instead of *t*11-18:1 (i.e. *trans*-10 shift) [4]. Avoiding the *trans*-10 shift is a main goal in the development of strategies to improve nutritional value of ruminant products since *t*10-18:1 is not a precursor of *c*9,*t*11-18:2 in tissues [1] and is considered as an unhealthy FA [5]. Moreover, in dairy ruminants, *trans*-10 shift has been related to the milk fat depression syndrome [6].

Global warming and climate changes concerns have been the trigger for intensive research on mitigating methane production by ruminants [7, 8]. Symbiotic relationships between rumen protozoa and methanogenic archea involving the H_2_ transfer from protozoa to methanogenese have been described. Defaunation (i.e. elimination of protozoa from rumen) is one of the strategies proposed to reduce methane emissions by ruminants [7, 8]. However, this approach may have implications in other mechanisms of rumen metabolism.

Ciliate protozoa, especially Entodiniomorphids, directly interfere on rumen metabolism of non-structural carbohydrates by engulfing the starch granules [9] and thus slow down starch fermentation and promote a more stable and higher rumen pH [10, 11]. Moreover, by predating amylolyticbacteria, ciliate protozoa also reduce the contribution of those bacteria to rumen starch digestion[9]. Thus, protozoa act as a stabilizing agent of starch fermentation in the rumen of grain-fed ruminants[8]. Besides that, Entodiniomorphids are also responsible for a direct reduction of lactic acid concentration, due to their high efficiency in removing the lactic acid from rumen environment [12] which also contributes to prevent pH values below 6.0, which is referred by [3] as the critical value for the activity of cellulolytic bacteria.

Rumen ciliate protozoa can comprise up to half of the rumen biomass and up to 75% of microbial lipids present in the rumen [9]. Their role on rumen lipid metabolism is still not totally understood [8], but it is generally accepted that ciliate protozoa are not directly involvedto RBH [2]. However, they actively contribute to lipolysis, namely the cellulolytic species as *Epidinium* sp. [13, 14] and influence the RBH by different mechanisms. Ciliate protozoa ingest and directly incorporate dietary PUFA in their cellular membranes, protecting them from hydrogenation by bacteria; they extensively prey and engulf several species of biohydrogenating bacteria and have symbiotic relationships with others [2, 10, 15–17].[18] reported that when compared with faunated lambs, protozoa-free lambs presented higher levels of SFA, including stearic acid (18:0) in the intramuscular fat (IMF). This may suggest that the presence of rumen protozoa may contribute to reduce RBH completeness (i.e. the proportion of dietary biohydrogenated UFA that end up as totally saturated FA) and consequently, the deposition of SFA in edible product. Moreover, ciliate membranes are rich in BI such as *t*11-18:1 and *c*9,*t*11-18:2[19, 20] and the levels of PUFA in protozoa cells are proportionally higher when compared to rumen bacteria [13, 15]. Despite the controversy over the mechanisms by which rumen protozoa accumulate *t*11-18:1, *c*9,*t*11-18:2 and PUFA in their membranes, due to their large contribution to rumen biomass, these microorganisms may represent an important reservoir of such FA.

Therefore, the main goal of the present work was to study the relationships between ciliate protozoa in rumen and the proportionsof the BI *t*10-18:1, *t*11-18:1, *c*9,*t*11-18:2, the occurrence of *trans*-10 shift, and the proportion of PUFA in rumen content and in meat of lambs fed with complete diets.

## Material and methods

### Animal experiments

Data used in the present study were obtained from three independent productive experiments performed with growing lambs, conducted by our research team in 2017 and 2018. All the experiments were performed at Estação Zootécnica Nacional, Instituto Nacional de Investigação Agrária e Veterinária (EZN-INIAV), located at Vale de Santarém, Portugal. They were conducted in the framework of the ValRuMeat research project to evaluate the impact of the diet on the nutritional value of meat from ruminants fattened in intensive production systems, particularly on the levels of *t*11-18:1 and *c*9, *t*11-18:2 in and the occurrence of the *trans*-10 shift.

A total of 86 lambs were used in the three experiments and all procedures followed the Directive 2010/63/EU which regulates the use of production animals in animal experiments, and were approved by the Organ Responsible for the Animal Welfare of INIAV I.P. (ORBEA-INIAV). The diets were formulated by our research group and prepared in the Feed Compound Unit of EZN-INIAV. Animal management, experimental conditions, slaughter, sample collection and analytical procedures were similar in the three experiments and were reported by [21]. The crossbred Merino Branco lambs, were reared by their dams on extensive pasture until weaning, with about 60 days of age. At that time, lambs were transported to EZN-INIAV, housed and randomly assigned to the individual pens and to the treatments. The diets were supplemented with 60 g/kg DM of soybean oil, to increase the supply of PUFA, mainly linoleic acid, in rumen. All the diets were presented in the ground form, except the diets with hay in Experiment 2. Feed was offered daily at 9:00 am at a rate of 110% of *ad libitum* intake and lambs had permanent access to clean water. Lambs were weighed every week and the duration of the trials was of 6 weeks, after 1 week of adaptation to the experimental conditions. In Table 1 are presented the summaries of the objectives and of the treatments in the three experiments. The detailed ingredient and chemical composition of the diets are presented in S1, S2 and S3 Tables.

**Table 1:** Brief description of the experiments whose data were used for the present study.

Treatments (Diets)	N	References
**1—Effect of NDF composition on rumen and lamb meat fatty acids composition**		
Increasing levels of alfalfa (Medicago sativa), were balanced by decreasing levels of soybean hulls (Glicine max) to formulate 3 diets with similar NDF content.		Santos-Silva et al. (2019)
**A20**—20% alfalfa	7	
**A40**—40% alfalfa	7	
**A60**—60% alfalfa	6	
**2—Effects of the physical form of alfalfa and the level of replacement in the diets of cereals by agro-industrial by-products on rumen and lamb meat fatty acid composition**		
Eight diets with a forage:concentrate ratio of 40:60 were formulated in a 2 × 4 factorial arrangement of treatments considering forage particle size and cereal replacement by Low Starch by-Products.		Submitted for publication
**AP0**—ground alfalfa and 0% barley grain	4	
**AP35**—ground alfalfa and 35% barley grain	4	
**AP65**—ground alfalfa and 65% barley grain	4	
**AP100**—ground alfalfa and 100% barley grain	4	
**AH0**—alfalfa hay and 0% barley grain	3	
**AH35**—alfalfa hay and 35% barley grain	4	
**AH65**—alfalfa hay and 65% barley grain	4	
**AH100**—alfalfa hay and 100% barley grain	4	
**3—Effects of the forage source and the proportion and type of dietary rumen buffer on rumen and lamb meat fatty acids composition**		
Five experimental diets with a forage:concentrate ratio of 40:60 were tested.		Unpublished results
**A0.5**—alfalfa and 0.5% of SB as buffer	7	
**A2.0**—alfalfa and 2.0% of SB as buffer	7	
**ALV**- alfalfa and 0.5% of SB plus 1.5% Levucell^®^ SC20 as buffer	6	
**R0.5**—ryegrass and 0.5% of SB as buffer	7	
**R2.0**—ryegrass and 2.0% of SB as buffer	7	

### Sample collection and laboratory analysis methods

Lambs had feed and water available until they were transported in groups of 3–4 to the experimental abattoir of EZN-INIAV, where they were slaughtered. Abattoir facilities are located at the campus of EZN-INIAV, about 400m from the lambs´ barn. Lambs waited a maximum of 30 minutes until they were stunned and exsanguinated.

Rumen content sampling was performed immediately after slaughter in the abattoir. The whole rumen content of each lamb was immediately collected, homogenized and a sample was strained through 4 layer of cheesecloth resulting in an aliquot with about 80 ml of rumen liquor. The pH of rumen content was immediately measured using a pH meter (Metrohm 744) and an aliquot of 2 ml was immediately preserved with 2 ml of 10% formalin solution and stored at 2°C until microscopic examination and analyses of the rumen ciliate protozoa. A representative sample of whole rumen content was also collected from each lamb and frozen, freeze-dried, milled and stored at −20°C until FA analysis. Also a sample of the liquid fraction of ruminal content was collected and frozen until volatile fatty acids analysis. *Longissimus thoracis* (Lt) muscle from all the carcasses were sampled at the third day after slaughter and the meat samples were frozen, freeze died and stored at −20°C until FA analysis, according to [22].

The analysis of ciliate protozoa was performed individually by microscopic counting as described by [23] and using a Brand^®^ counting chamber Blaubrand^®^ Neubauer Improved with 0·100 mm depth (BR7178110 Sigma-Aldrich, Portugal) and a LeitzLaborlux K binocular light microscope (Leitz, Germany). Ciliate cell numbers were determined in duplicate for each sample and the identification at the genus level was made based on protozoa morphology, according to [24]. Protozoa community structure was also accessed by the type of rumen protozoa population (A B or O), according to [24]. Microscopic counting of protozoa was performed since it allows a better accuracy when relating the rumen concentration of total ciliates and of individual genera with rumen parameters and meat FA[8].

Fatty acid methyl esters (FAME) from freeze-dried rumen content samples were directly transesterified by reaction with sodium methoxide in methanol (0.5 M) at 50°C for 10 min followed by reaction with HCl in methanol (2.5 M) at 80°C for 15 min. Afterwards, thin-layer chromatography was used to separate FAME (fraction 1) from the fractions containing oxo- and hydroxy-groups and dimethylacetals (fraction2) as described by [25]. Methyl heneicosanoate (21:0) (internal standard) was used for quantification at concentrations: 1 mg for FAME and 50 g for oxo- and hydroxy-FAME. FAME were quantified by GC with flame ionization detection (GC-FID) using a Shimadzu GC 2010-Plus (Shimadzu, Kyoto, Japan) equipped with aSLB-IL111 (100 m × 0.25 mm, 0.20 m film thickness, Supelco, Bellefonte, PA, USA) capillary column. The chromatographic conditions were as follow: injector and detector temperatures were set at 250°C, helium was used as the carrier gas at1 ml/min constant flow and two different oven temperature programs were used to analyze the two fractions. For the FAME fraction, the GC oven was maintained at 168°C for 43 min, afterwards the temperature was increased at 2°C/min to 220°C, and kept at this temperature for 10 min.

Intramuscular lipids were extracted from Lt muscle as described by [26]. Fatty acids were transesterified according to[27], using sodium methoxide in methanol, followed by hydrochloric acid in methanol (1:1 v/v). Fatty acid methyl esters were analyzed using a Shimadzu GC2010Plus chromatograph (Shimadzu, Kyoto, Japan), equipped with a flame-ionization detector and fused silica capillary column (SP-2560 (100 m × 0.25 mm internal diameter × 0.20 μm film thickness, Supelco, Bellefonte, PA, USA)). The injector and detector temperatures were 250°C and 280°C, respectively. The initial oven temperature of 50°C was held for 1 min, increased at 50 °C/min to 150°C and held for 20 min, increased at 1°C/min to 190°C and then increased at 2°C/min to 220°C and held for 40 min. Helium was used as carrier gas at a flow rate of 1 ml/min and the split ratio was 50:1. Nonadecanoic acid (19:0) was used as internal standard to quantify muscle lipid FA methyl esters. Fatty acids were identified by comparison of the FAME retention times with those of authentic standards (FAME mix 37 components from Supelco Inc., Bellefont, PA, USA) and by comparison with published chromatograms [28, 29].

### Calculations

The biohydrogenation completeness (BC) (%), reports the extent of the biohydrogenation of dietary *c*9-18:1, 18:2 n-6 and 18:3 n-3 and was estimated considering the maximum 18:0 content in rumen, assuming a complete biohydrogenation of the C18 FA from diet as proposed by [30]. The calculations were as follow:

$$BC\left(\%\right)=\left(\frac{18:0r}{\left(c9,18:1d-c9,18:1r\right)+\left(18:2n6d-18:2n6r\right)+\left(18:3n3d-18:3n3r\right)+18:0d}\right)x100$$

where 18:0r, is the 18:0 in rumen as percentage of total C18 FA; *c*9,18:1d/r is the *c*9-18:1 in diet or rumen as percentage of total C18 FA; 18:2n6d/r: is the 18:2n−6 in diet or rumen as percentage of total C18 FA;18:3n3d/r is the 18:3n−3 in diet or rumen as percentage of total C18 FA; 18:0d is the 18:0 in diet as percentage of total C18 FA.

### Statistical analysis

Initially, using a pool of the three experiments (n = 86 individual data), it was performed a global Spearman correlation analysis to test the existence of relationships between the concentrations (log_10_ cells/ml rumen liquor) of: 1) Total Ciliates; 2) Holotrichs (ciliates from the Order Vestibuliferida); 3) Entodiniomorphids (ciliates from de Order Entodiniomorphida) and 4) individual genera of ciliates that occurred in more than 15% of the rumen samples (*Isotricha*, *Dasytricha*, *Entodinium*, *Polyplastron*, *Diplodinium* and *Epidinium*) with: 1) FA proportions in rumen content; 2) rumen pH and 3) FA proportions in intramuscular fat (IMF) of Lt muscle. The Spearman rank order correlation coefficients were determined with STATISTICA^®^10software (StatSoft Inc., 2010).

In addition and in order to confirm if the relationships found with the correlation analysis were maintained when the experiment was considered in the model, a linear regression analysis was performed using the SAS MIXED procedure (SAS Institute Inc. Cary, NC). The relationships between concentration (as log_10_ cells/ml) of rumen ciliate protozoa (Total ciliates, Holotrichs, Entodiniomorphids and ciliate genera presented in more than 30% of rumen samples: *Isotricha*, *Entodinium* and *Epidinium*) and the FA in rumen and Lt muscle were evaluated with a linear model that included the concentration of ciliate protozoa as fixed effects and the experiment as a discrete random effect. An analysis of variance to evaluate the effect of the type of protozoa population in rumen and Lt muscle FA was performed, using the MIXED procedure of SAS, and when the effect of protozoa population was significant, the least square means were compared, using a pairwise Tukey’s comparison test. In statistical models, the variance was accommodated in the model using the covariance structure with the best fit to the data. Statistical significance was set for a level of *P*< 0.05, but regression parameters for models with 0.05≤*P*≤0.06 were also considered.

## Results

### Rumen ciliate protozoa densities

**Fig 1:**
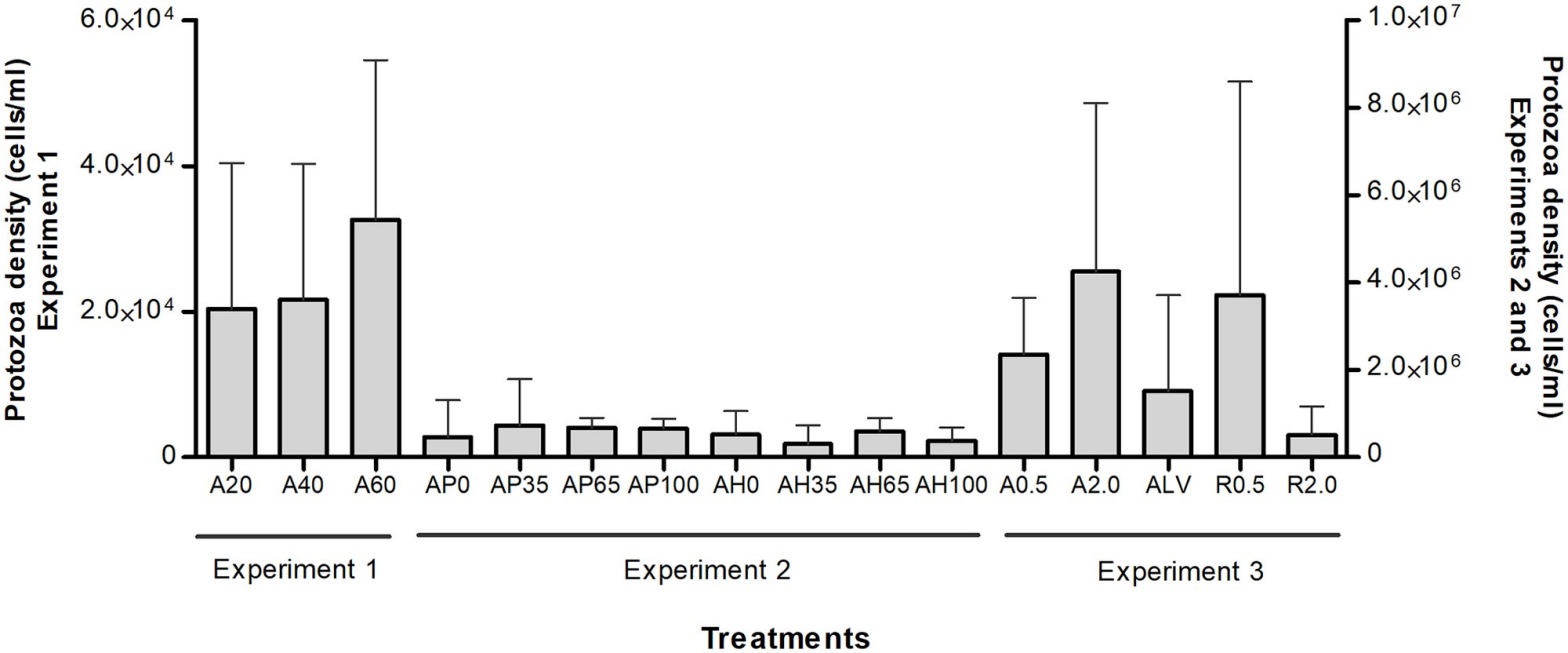
Total density of rumen ciliate protozoa. Total density of ciliates (arithmetic mean and standard deviation) in the 86 lambs rumen samples, grouped by treatments and by the experiments.

**Fig 2:**
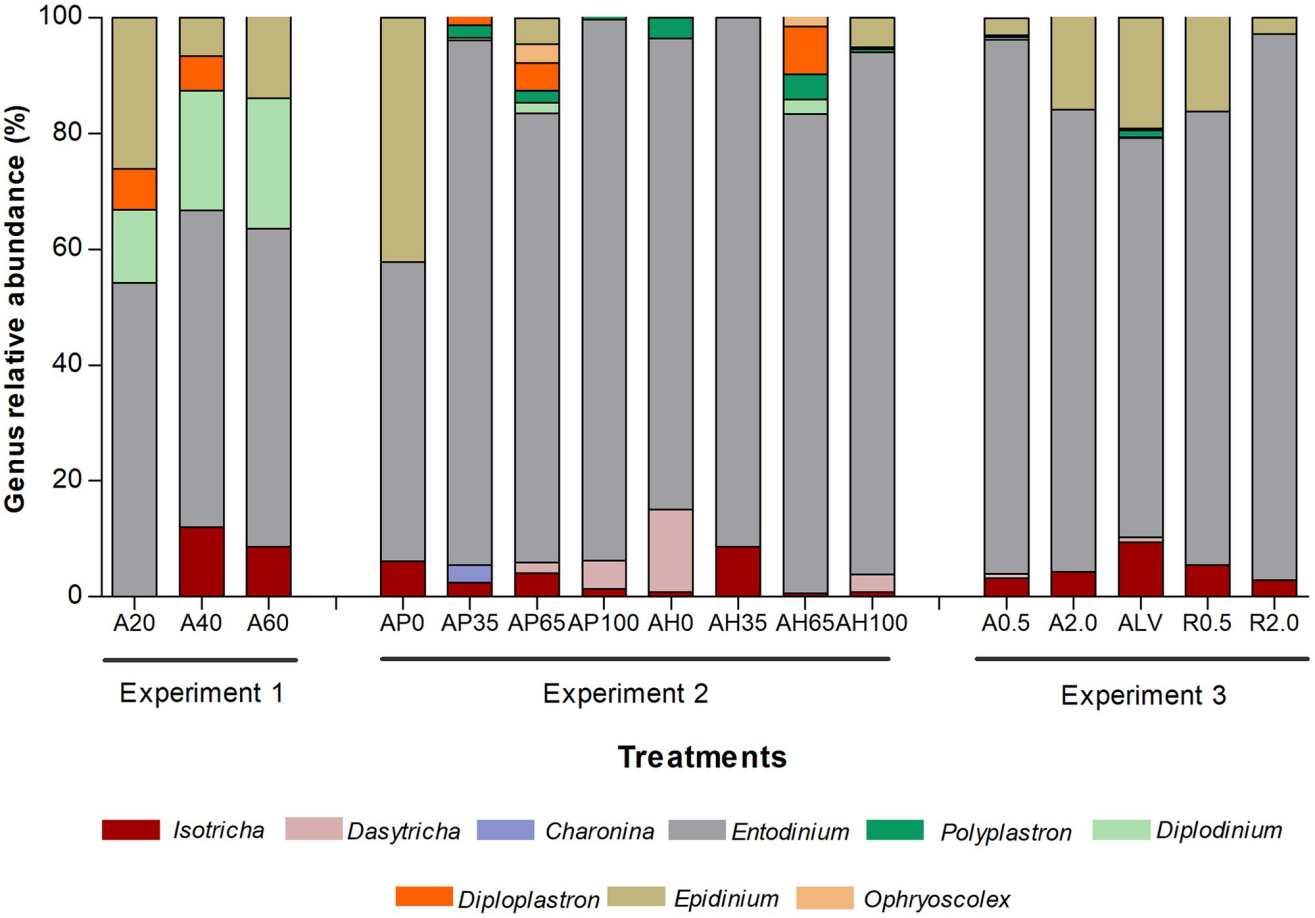
Relative abundance of each ciliate genus. Relative abundances for each ciliate genus identified in the 86 lambs rumen samples grouped by treatments and by experiments.

The individual results on total ciliate protozoa abundance and on ciliate genus relative abundance are presented in Figs 1 and 2, respectively. From the total of 86 lambs sampled in the study, 10 of them (n = 4 in Experiment 1, n = 3 in Experiment 2 and n = 3 in Experiment 3) did not present protozoa cells in the rumen liquor samples (0 cells/ml rumen liquor) and therefore were considered as defaunated. The remaining lambs presented, in average, 1.35 × 10^6^ ciliates per ml of rumen fluid, and a large individual variability was observed among lambs (Fig 1).

Nine ciliate genera were identified: three Holotrichs (*Isotricha*, *Dasytricha and Charonina)* and six Entodiniomorphids (*Entodinium*, *Diplodinium*, *Polyplastron*, *Diploplastron*, *Epidinium* and *Ophryoscolex)* (Fig 2). Entodiniomorphids were observed in the rumen of all faunated lambs (n = 76), and represented 95% of total ciliates present. Globally, the amilolytic genus *Entodinium* was the most abundant, averaging 1.17 × 10^6^cells / ml (n = 71 lambs), followed by the celulolytic *Epidinium* (5.34 × 10^5^cells / ml) (n = 23 lambs), and the mainly soluble sugars consumer *Isotricha* (1.30× 10^5^cells / ml) (n = 36 lambs). The least abundant genera were *Ophyroscolex* (6.50 × 10^3^ cells / ml) (n = 2 lambs) and *Diplodinium* (2.01× 10^4^ cells / ml) (n = 14 lambs). The distribution of ciliates genera also presented a high variability, even for lambs fed the same diet (Fig 2).

### Characterization of rumen variables and muscle FA

The mean, standard deviation, minimum and maximum values obtained in each one of the three experiments whose data was used and for the variables considered for correlation and regression analyses (rumen ciliate protozoa concentration, total FA content and FA proportions of rumen content, RBH completeness, rumen pH, FA content and FA proportions of Lt muscle) are presented in Tables 2–4 for Experiments 1, 2 and 3, respectively. Santos-Silva, Francisco (21) reported the results concerning the effect of the diets of Experiment 1 on Lt muscle fatty acids. The detailed effect of diets in rumen and muscle FA, in rumen pH and in RBH completeness for Experiments 2 and 3 will be published elsewhere.

**Table 2:** Mean, standard deviation, minimum and maximum values for the phenotypes identified in Experiment 1.

	Rumen				*Longissimus thoracis* muscle			
	Mean	s.d	Max	Min	Mean	s.d	Max	Min
Total ciliates (log_10_ cells/ml rumen liquor)	3.52	1.826	4.80	0.00				
Holotrichs	0.42	1.305	4.26	0.00				
*Isotricha*	0.42	1.305	4.26	0.00				
*Dasytricha*	0.00	0.000	0.00	0.00				
Entodiniomorphids	3.50	1.815	4.80	0.00				
*Entodinium*	2.95	1.993	4.56	0.00				
*Diplodinium*	1.43	2.006	4.35	0.00				
*Polyplastron*	0.00	0.000	0.00	0.00				
*Diploplastron*	0.40	1.225	4.00	0.00				
*Epidinium*	1.22	1.916	4.26	0.00				
Fatty acids (FA) (% total FA)								
18:0	35.0	6.59	47.6	25.2	16.0	1.60	14.0	20.0
*c*9-18:1	10.5	1.13	13.1	7.98	31.9	2.24	35.2	27.3
18:2 n-6	9.86	2.881	15.0	5.27	8.27	1.648	11.27	6.10
18:3 n-3	1.30	0.348	1.92	0.74	0.63	0.109	0.886	0.480
*t*10-18:1	11.0	9.20	30.0	1.18	4.51	2.168	8.99	1.42
*t*11-18:1	5.71	5.064	16.5	1.08	1.60	0.847	3.76	0.544
*t*10/*t*11 ratio	5.72	6.975	21.7	0.15	4.38	4.138	15.26	0.576
*c*9,*t*11-18:2	0.32	0.296	0.95	0.02	0.55	0.269	1.200	0.176
*t*10,*c*12-18:2	0.44	0.278	1.05	0.05	0.064	0.0344	0.142	0.015
18:1 BI	22.4	7.19	37.4	11.8				
18:2 BI	12.8	1.32	15.4	9.78				
SFA	49.7	6.58	62.3	38.1	42.5	1.64	44.72	39.26
*cis*-MUFA					34.4	2.38	38.3	29.4
n-6 PUFA					9.81	0.986	13.70	7.39
n-3 PUFA					1.07	0.167	1.45	0.84
n-6 LC-PUFA					1.36	0.400	2.22	0.83
n-3 LC-PUFA					0.43	0.096	0.66	0.31
PUFA	11.9	3.25	17.7	6.7	11.03	2.13	15.26	8.42
Total FA (mg/g DM)	76.7	13.07	88.3	29.5	103.8	26.98	58.12	149.80
RBH completeness (%)	60.1	11.50	78.4	39.9				
Rumen pH	5.57	0.272	6.19	5.29				

RBH, Rumen biohydrogenation; BI, Biohydrogenation intermediates; SFA, Saturated fatty acids; *cis*-MUFA, *cis* monosaturated fatty acids; PUFA, Polyunsaturated fatty acids; n-6 PUFA, 18:2 n-6 + 20:4 n-6 + 22:4 n-6; LC n-6 PUFA, 20:4 n-6 + 22:4 n-6; n-3 PUFA, 18:3 n-3 + 20:5 n-3 + 22:5 n-3 + 22:6 n-3; LC n-3 PUFA, 20:5 n-3 + 22:5 n-3 + 22:6 n-3.

**Table 3:** Mean, standard deviation, minimum and maximum values for the phenotypes identified in Experiment 2.

	Rumen				*Longissimus thoracis* muscle			
	Mean	s.d	Max	Min	Mean	s.d	Max	Min
Total ciliates (log_10_ cells/ml rumen liquor)	5.24	1.500	6.36	0.00				
Holotrichs	2.58	2.316	5.48	0.00				
*Isotricha*	1.91	2.240	5.05	0.00				
*Dasytricha*	1.07	1.994	5.48	0.00				
Entodiniomorphids	5.21	1.496	6.33	0.00				
*Entodinium*	5.17	1.480	6.31	0.00				
*Diplodinium*	0.69	1.591	4.49	0.00				
*Polyplastron*	1.16	1.964	4.87	0.00				
*Diploplastron*	1.07	1.984	5.07	0.00				
*Epidinium*	0.53	1.621	5.89	0.00				
Fatty acids (FA)(% total FA)								
18:0	27.1	9.58	45.9	8.98	15.9	1.41	19.1	12.7
*c*9-18:1	11.4	2.76	17.8	6.3	31.1	2.61	37.2	24.5
18:2 n-6	12.6	6.06	25.9	3.6	7.01	1.559	10.70	4.06
18:3 n-3	1.60	0.656	3.02	0.47	0.65	0.117	0.864	0.457
*t*10-18:1	2.21	2.150	11.0	0.80	1.77	1.123	5.06	0.43
*t*11-18:1	14.6	5.90	23.5	4.8	4.69	1.718	9.07	2.28
*t*10/*t*11 ratio	0.21	0.243	0.98	0.04	0.449	0.420	1.520	0.121
*c*9,*t*11-18:2	0.33	0.222	0.97	0.06	1.42	0.382	2.35	0.75
*t*10,*c*12-18:2	0.08	0.0736	0.31	0.00	0.00	0.000	0.00	0.00
18:1 BI	24.0	5.00	33.1	14.8				
18:2 BI	1.52	0.578	3.92	0.81				
SFA	45.3	10.55	65.5	26.1	41.6	1.56	44.5	38.1
*cis*-MUFA					34.1	2.72	40.03	27.12
n-6 PUFA					8.80	1.866	13.40	5.02
n-3 PUFA					0.82	0.148	1.11	0.56
n-6 LC-PUFA					1.62	0.443	2.47	0.87
n-3 LC-PUFA					0.44	0.135	0.75	0.24
PUFA	14.6	6.71	28.6	4.27	10.0	2.03	14.8	5.83
Total FA (mg/g DM)	88.3	14.59	114	56.7	93.8	23.87	150.6	59.2
RBH completeness (%)	51.7	13.44	74.1	25.9				
Rumen pH	5.90	0.441	6.89	5.25				

RBH, Rumen biohydrogenation; BI, Biohydrogenation intermediates; SFA, Saturated fatty acids; *cis*-MUFA, *cis* monosaturated fatty acids; PUFA, Polyunsaturated fatty acids; n-6 PUFA, 18:2 n-6 + 20:4 n-6 + 22:4 n-6; LC n-6 PUFA, 20:4 n-6 + 22:4 n-6; n-3 PUFA, 18:3 n-3 + 20:5 n-3 + 22:5 n-3 + 22:6 n-3; LC n-3 PUFA, 20:5 n-3 + 22:5 n-3 + 22:6 n-3.

**Table 4:** Mean, standard deviation, minimum and maximum values for the phenotypes identified in Experiment 3.

	Rumen				*Longissimus thoracis* muscle			
	Mean	s.d	Max	Min	Mean	s.d	Max	Min
Total ciliates (log_10_ cells/ml rumen liquor)	5.43	2.022	7.09	0.00				
Holotrichs	2.96	2.565	5.87	0.00				
*Isotricha*	2.95	2.553	5.87	0.00				
*Dasytricha*	0.40	1.341	5.16	0.00				
Entodiniomorphids	5.26	2.206	7.09	0.00				
*Entodinium*	4.88	2.450	1.05	0.00				
*Diplodinium*	0.24	1.032	4.84	0.00				
*Polyplastron*	0.39	1.325	4.78	0.00				
*Diploplastron*	0.12	0.746	4.48	0.00				
*Epidinium*	2.25	2.866	6.27	0.00				
Fatty acids (FA)(% total FA)								
18:0	41.9	11.44	65.0	21.8	16.1	1.63	19.1	12.4
*c*9-18:1	6.12	2.111	10.1	2.87	31.1	2.51	36.2	25.6
18:2 n-6	4.07	1.861	7.77	1.43	6.85	1.650	11.17	4.48
18:3 n-3	0.60	0.261	1.50	0.31	0.42	0.111	0.85	0.30
*t*10-18:1	2.32	4.428	24.0	0.51	2.32	1.466	7.37	0.50
*t*11-18:1	11.4	5.14	21.7	3.56	4.05	1.694	8.28	1.74
*t*10/*t*11 ratio	0.30	0.749	4.33	0.03	0.78	0.846	4.22	0.11
*c*9,*t*11-18:2	0.41	0.313	1.45	0.06	1.17	0.453	2.14	0.55
*t*10,*c*12-18:2	0.00	0.00	0.00	0.00	0.00	0.000	0.00	0.00
18:1 BI	19.0	5.99	35.4	9.90				
18:2 BI	1.16	0.519	2.76	0.33				
SFA	63.0	8.23	80.5	50.2	43.0	1.89	47.4	39.5
*cis*-MUFA					35.4	2.50	40.7	30.1
n-6 PUFA					8.79	2.058	14.2	5.88
n-3 PUFA					0.76	0.166	1.20	0.50
n-6 LC-PUFA					1.89	0.514	3.14	1.15
n-3 LC-PUFA					0.35	0.104	0.60	0.20
PUFA	4.68	2.017	8.84	1.78	9.57	2.218	15.4	6.43
Total FA (mg/g DM)	72.7	23.68	133.5	18.7	92.3	19.93	130.1	52.1
RBH completeness (%)	68.3	10.47	87.7	46.0				
Rumen pH	6.83	0.402	7.767	6.10				

RBH, Rumen biohydrogenation; BI, Biohydrogenation intermediates; SFA, Saturated fatty acids; *cis*-MUFA, *cis* monosaturated fatty acids; PUFA, Polyunsaturated fatty acids; n-6 PUFA, 18:2 n-6 + 20:4 n-6 + 22:4 n-6; LC n-6 PUFA, 20:4 n-6 + 22:4 n-6; n-3 PUFA, 18:3 n-3 + 20:5 n-3 + 22:5 n-3 + 22:6 n-3; LC n-3 PUFA, 20:5 n-3 + 22:5 n-3 + 22:6 n-3.

Globally, it was observed a high variation for each phenotype among the three experiments and the 16 diets used. In Experiment 1, were observed the highest values for *t*10-18:1, *t*10,*c*12-18:2 and *t*10/*t*11 ratio and the lowest values for *t*11-18:1 and *c*9,*t*11-18:2. In Experiment 3 were observed the highest values for 18:0 and RBH completeness and the lowest values for 18:2 n-6, 18:3 n-3 and PUFA. In Experiment 2 were observed the highest values for rumen *c*9-18:1 and for rumen and muscle 18:3 n-3, and intermediate values for the other phenotypes. The lowest values for rumen pH were observed in Experiment 1 and the highest in Experiment 3.

### Correlations between ciliate protozoa and rumen variables and *longissimus thoracis* muscle fatty acids

**Fig 3:**
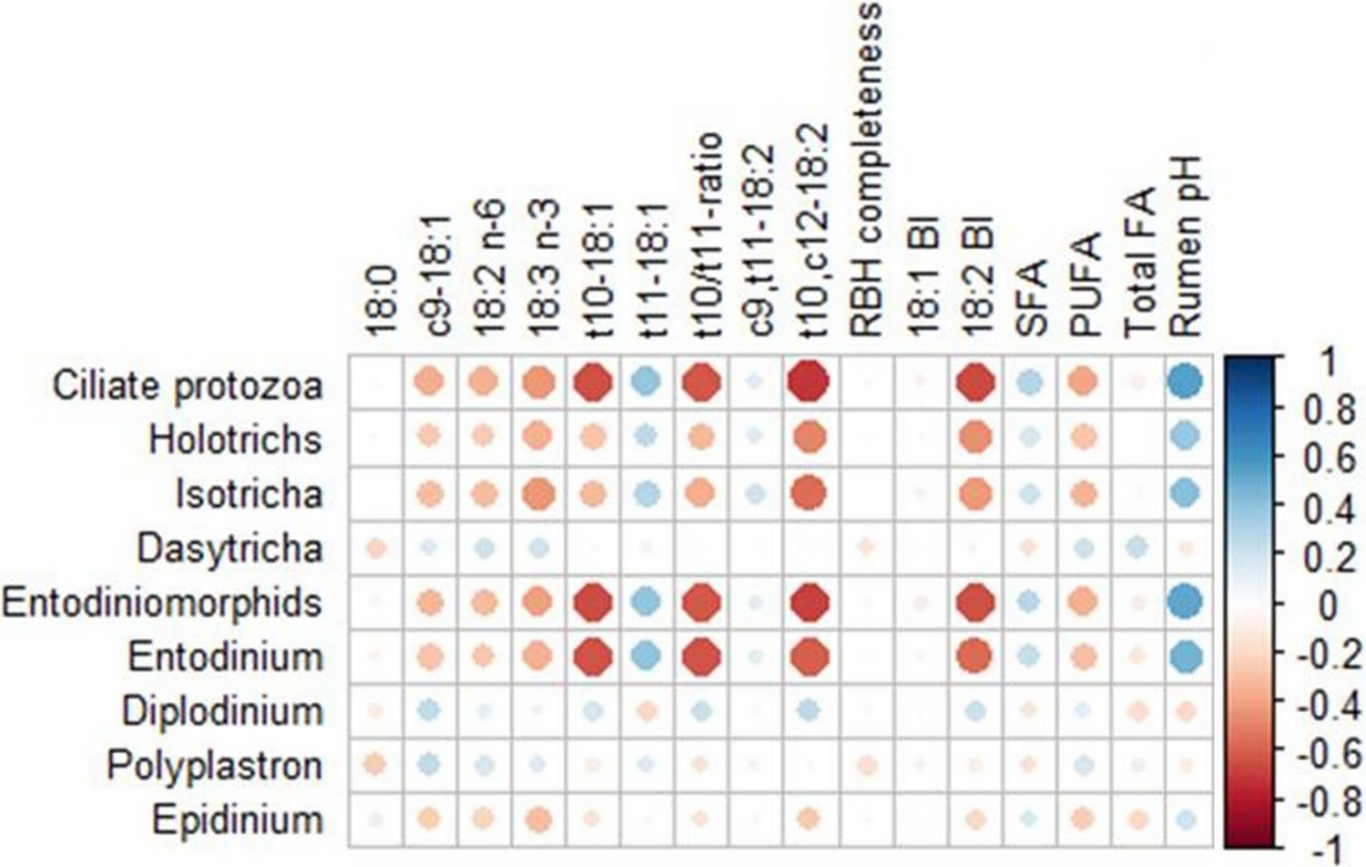
Significant correlations among ciliates (log_10_cells/ml) and rumen fatty acids (g/100g FA), biohydrogenation completeness (%) and rumen pH. The strongest Spearman´scorrelations are represented by large circles, while the weakest correlations are represented by small circles. The colors and their intensity on the bar scale denote the nature of the correlation, with the darker shade of blue indicating more positive correlations (close to 1) and the darker shade of red indicating more negative correlations (closer to -1) (n = 86). RBH, Rumen biohydrogenation; BI, Biohydrogenation intermediates; SFA, Saturated fatty acids; PUFA, Polyunsaturated fatty acids.

The significant Spearman´s rank correlations between ciliate protozoa concentration (as log_10_cells/ml), rumen FA and pH and, biohydrogenation completeness, performed with a pool of the data from the three experiments, are shown in Fig 3. Several moderate and strong correlations, between -0.71 and 0.55, were observed between protozoa concentration and FA composition of the whole rumen content and rumen pH. Total ciliates were mainly negatively correlated with the *t*10,*c*12- 18:2 (ρ = -0.71; *P* < 0.001), 18:2 BI (ρ = -0.65; *P* < 0.001), *t*10-18:1 (ρ = -0.64; *P*< 0.001) and *t*10/*t*11 ratio (ρ = -0.61; *P*< 0.001). Holotrichs showed the strongest negative correlations with *t*10,*c*12- 18:2 (ρ = -0.48; *P* < 0.001), 18:2 BI (ρ = -0.47; *P* < 0.001), 18:3 n-3 (ρ = -0.35; *P* = 0.002) and *t*10/*t*11 ratio (ρ = -0.31; *P* = 0.006) and Entodiniomorphids mainly negatively correlated with *t*10,*c*12- 18:2 (ρ = -0.68; *P* < 0.001), *t*10-18:1(ρ = -0.64; *P* < 0.001), 18:2 BI (ρ = -0.63; *P* < 0.001) and *t*10/*t*11 ratio (ρ = -0.62; *P* < 0.001). Concerning the ciliates individual genera, the strongest negative correlations were observed between *Entodinium* and t10/t11 ratio (ρ = -0.63; *P* < 0.001), *t*10-18:1 (ρ = -0.62; *P* < 0.001), *t*10,*c*12-18:2 (ρ = -0.60; *P* < 0.001), and 18:2 BI (ρ = -0.57; *P* < 0.001) and between *Isotricha* and *t*10,*c*12-18:2 (ρ = -0.56; *P* < 0.001).

Total ciliates were mainly positively correlated with rumen pH (ρ = +0.55; *P* < 0.001) and with *t*11-18:1 (ρ = +0.38; *P* < 0.001). As observed for the total ciliates, both Holotrichs and Entodiniomorphids showed the highest positive correlations with rumen pH (ρ = +0.37; *P* < 0.001 and ρ = +0.52; *P* < 0.001, respectively) and with *t*11-18:1 (ρ = +0.25; *P* = 0.027 and ρ = +0.38; *P*< 0.001, respectively). In relation to ciliates individual genera, the strongest positive correlations were observed between *Entodinium* and rumen pH (ρ = +0.46; *P* < 0.001), *Isotricha* and rumen pH (ρ = +0.41; *P* < 0.001) and *Entodinium* and *t*11-18:1 (ρ = +0.40; *P* < 0.001).

**Fig 4:**
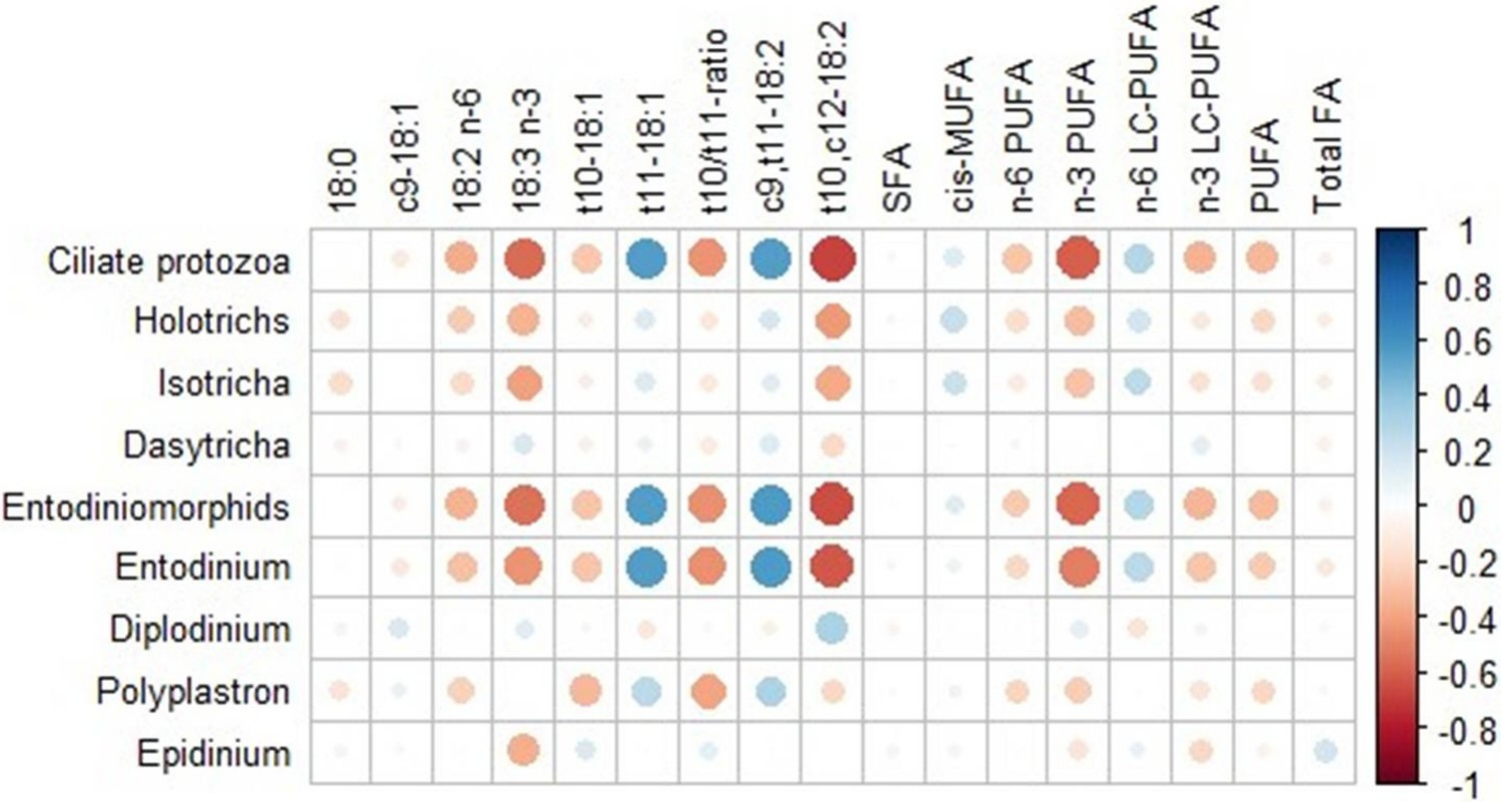
Significant correlations among ciliates (log_10_cells/ml) and fatty acids (g/100g FA) of *longissimus* muscle of lambs. The strongest Spearman´s correlations are represented by large circles, while the weakest correlations are represented by small circles. The colors and their intensity on the bar scale denote the nature of the correlation, with the darker shade of blue indicating more positive correlations (close to 1) and the darker shade of red indicating more negative correlations (closer to -1) (n = 86). SFA, Saturated fatty acids; *cis*-MUFA, *cis* monosaturated fatty acid; PUFA, Polyunsaturated fatty acids; n-6 PUFA, 18:2 n-6 + 20:4 n-6 + 22:4 n-6;LC n-6 PUFA, 20:4 n-6 + 22:4 n-6; n-3 PUFA,18:3 n-3 + 20:5 n-3 + 22:5 n-3 + 22:6 n-3; LC n-3 PUFA, 20:5 n-3 + 22:5 n-3 + 22:6 n-3.

Results concerning the correlation analysis among ciliates concentration in rumen and the FA present in Lt muscle of the 86 lambs are shown in Fig 4. In general, the correlations that were observed between protozoa and FA in rumen were also observed with the same FA in muscle, but with lower coefficients. Several moderate and strong correlations, between -0.67 and 0.56, were observed between rumen ciliates and the muscle FA in study. Total ciliates were mainly negatively correlated with *t*10,*c*12-18:2 (ρ = -0.68; *P* < 0.001), n-3 PUFA (ρ = -0.60; *P* < 0.001), 18:3 n-3 (ρ = -0.57; *P* < 0.001) and *t*10/*t*11 ratio (ρ = -0.45; *P* < 0.001). Holotrichs had the strongest negative correlations with and *t*10,*c*12- 18:2 (ρ = -0.42; *P* < 0.001), 18:3 n-3 (ρ = -0.35; *P* = 0.002) and n-3 PUFA (ρ = -0.30; *P* = 0.007) and Entodiniomorphids were mainly negatively correlated with *t*10,*c*12- 18:2 (ρ = -0.65; *P* < 0.001), n-3 PUFA (ρ = -0.58; *P* < 0.001), 18:3 n-3 (ρ = -0.54; *P* < 0.001) and *t*10/*t*11 ratio (ρ = -0.45; *P* < 0.001). Considering ciliates individual genera, the strongest negative correlations were observed between *Entodinium* and *t*10,*c*12- 18:2 (ρ = -0.61; *P* < 0.001), n-3 PUFA (ρ = -0.51; *P* < 0.001), *t*10/*t*11 ratio (ρ = -0.46; *P* < 0.001) and 18:3 n-3 (ρ = -0.45; *P* < 0.001), between *Polyplastron* and *t*10/*t*11 ratio (ρ = -0.40; *P* < 0.001) and *Isotricha* and 18:3 n-3 (ρ = -0.40; *P*< 0.001) and *t*10,*c*12-18:2 (ρ = -0.37; *P* < 0.001). The strongest positive correlations between total ciliates and muscle FA were observed with *t*11-18:1 (ρ = +0.55; *P* < 0.001) and with *c*9,*t*11-18:2 (ρ = +0.55; *P* < 0.001). Also for Entodiomorphids these were the main positive correlations observed (ρ = +0.56; *P* < 0.001, for both *t*11-18:1 and *c*9,*t*11-18:2). For Holotrichs, only a significant and positive correlation was found, and it was between the individual genus, *Isotricha* and n-6 LC-PUFA (ρ = +0.25; *P* = 0.029). *Entodinium* was the ciliate genus that strongly correlated with the muscle FA in study. The highest positive correlations were observed between *Entodinium* and *c*9,*t*11-18:2 (ρ = +0.56; *P* < 0.001) and *t*11-18:1 (ρ = +0.55; *P* < 0.001).

### Regression analysis between ciliates concentration, rumen variables and meat fatty acids

Tables 5 and 6 show the regression coefficients with significant effect for rumen ciliates and rumen and meat FA, obtained with models that included the experiment as a random effect. In rumen (Table 5), the 18:0 (*P* = 0.015), the *t*10-18:1 (*P* = 0.058), the *t*10/*t*11 ratio (*P* = 0.002) and the 18:2 BI (*P* = 0.021) decreased linearly with the concentration of Total ciliates. Stearic acid (18:0) (*P* = 0.020), 18:2 BI (*P* = 0.002), SFA (*P* = 0.011) and RBH completeness (*P* = 0.029) decreased linearly with Holotrichs and 18:0 (*P* = 0.010), *t*10-18:1 (*P* = 0.062), *t*10/*t*11 ratio (*P* < 0.001) and RBH completeness (*P* = 0.029) decrease linearly with Entodiniomorphids. At the genus level, it were also observed linear decreases of 18:0 (*P* = 0.014), 18:2 BI (*P* = 0.016), SFA (*P* = 0.054) and RBH completeness (*P* = 0.011) with *Isotricha*, of *t*10/*t*11 ratio (*P* < 0.001) with *Entodinium* and of Total FA (*P* = 0.020), *c*9-18:1 (*P* < 0.001), *t*10-18:1 (*P* = 0.062), *t*10/*t*11 ratio (*P* < 0.001), 18:2 BI (*P* = 0.016) and RBH completeness (*P* = 0.027) with *Epidinium*. Linear increases of the *t*11-18:1 (*P* <0.001) and SFA (*P* <0.001) were observed for Total ciliates in rumen. The *t*11-18:1 also increased linearly with Entodiniomorphids (*P* = 0.011), *Isotricha* (*P* <0.001), *Entodinium* (*P* = 0.018) and *Epidinium* (*P* = 0.023). Linear increases of *c*9,*t*11-18:2 with *Isotricha* (*P* = 0.041), of 18:1 BI with *Isotricha* (*P* = 0.017), *Entodinium* (*P* = 0.003) and *Epidinium* (*P* = 0.004) and of rumen pH with Holotrichs (*P* = 0.025) were also observed.

**Table 5:** Significant linear regression between rumen ciliates (log_10_cells/ml) and rumen variables.

Rumen variables		Regression equations	*P*value
Fatty acids (g/100g FA)	Total FA	83.3±5.12–1.66±0.695 ×*Epidinium*^1^	0.020
	18:0	61.0±11.09–4.70±1.885 × Total ciliates^1^	0.015
		37.6±3.94–1.10±0.463 × Holotrichs^1^	0.020
		37.3±4.14–1.15±0.459 ×*Isotricha*^1^	0.014
		64.5±11.99–5.40±2.034 × Entodiniomorphids^1^	0.010
	*c*9-18:1	9.39±1.148–0.057±0.0136 × *Epidinium*^1^	<0.001
	*t*10-18:1	3.78±1.203–0.289±0.1496 × Total ciliates^1^	0.058
		4.88±2.468–0.243±0.1281 × Entodiniomorphids^1^	0.062
		2.27±1.037–0.049±0.0257 × *Epidinium*^1^	0.062
	*t*11-18:1	-9.97±6.023 + 3.92±1.058 × Total ciliates^1^	<0.001
		8.81±2.620 + 1.04±0.260 × *Isotricha*^1^	<0.001
		-4.69±6.367 + 2.94±1.125 × Entodiniomorphids^1^	0.011
		6.16±3.021 + 0.983±0.4066 × *Entodinium*^1^	0.018
		10.5±2.85 + 0.498±0.2137 × *Epidinium*^1^	0.023
	*t*10/*t*11 ratio	0.604±0.2378–0.053±0.0163 × Total ciliates^1^	0.002
		0.436±0.0708–0.056±0.0118 × Entodiniomorphids^1^	<0.001
		0.261±0.0426–0.028±0.072 × *Entodinium*^1^	<0.001
		0.114±0.0107–0.011±0.022 × *Epidinium*^1^	<0.001
	*c*9,*t*11-18:2	0.290±0.0345 + 0.022±0.0104 × *Isotricha*^1^	0.041
	18:1 BI	20.2±2.21 + 0.661±0.2717 × *Isotricha*	0.017
		15.8±3.19 + 1.25±0.408 × *Entodinium*^1^	0.003
		20.4±2.14 + 0.580±0.1938 × *Epidinium*^1^	0.004
	18:2 BI	6.24±3.654–0.235±0.1000 × Total ciliates^1^	0.021
		5.11±3.650–0.051±0.0160 × Holotrichs^1^	0.002
		5.08±3.662–0.042±0.0168 × *Isotricha*^1^	0.016
		4.87±3.713–0.045±0.0150 × *Epidinium*^1^	0.004
	SFA	27.0±5.25+ 4.76±0.778 ×Total ciliates^1^	<0.001
		55.6±4.24–0.924±0.355 × Holotrichs^1^	0.011
		54.8±4.77–0.76±0.389 × *Isotricha*^1^	0.054
RBH completeness (%)		63.5±6.36–1.11±0.497 × Holotrichs^1^	0.029
		63.7±6.49–1.29±0.491 × *Isotricha*^1^	0.011
		86.9±13.73–4.82±2.161 × Entodiniomorphids^1^	0.029
		62.3±6.45–1.20±0.531 × *Epidinium*^1^	0.027
Rumen pH		5.97±0.376 + 0.040±0.0174 × Holotrichs^1^	0.025

SFA, Saturated fatty acids; BI, Biohydrogenation intermediates; RBH, Rumen biohydrogenation.

^1^log_10_ cells/ml

**Table 6:** Significant linear regression equations between rumen ciliates (log_10_cells/ml) and meat FA.

Meat fatty acids (g/100g FA)	Regression equations	*P*value
18:0	19.0±1.09–0.522±0.1795 × Entodiniomorphids^1^	0.005
*c*9-18:1	33.6±1.13–0.420±0.2008× Total ciliates^1^	0.040
	31.5±0.16–0.096±0.0448 ×Holotrichs^1^	0.035
	31.5±0.14–0.100±0.0401 × *Isotricha*^1^	0.014
	33.8±0.96–0.458±0.1700 × Entodiniomorphids^1^	0.009
	34.1±1.13–0.536±0.2050 × *Entodinium*^1^	0.011
	32.5±0.20–0.266±0.0631 × *Epidinium*^1^	<0.001
18:2 n-6	9.97±0.848–0.542±0.1588 ×Total ciliates^1^	0.001
	11.1±1.02–0.732±0.1825 × Entodiniomorphids^1^	<0.001
	9.43±0.752–0.452±0.1447 × *Entodinium*^1^	0.003
18:3 n-3	0.593±0.874–0.011±0.0033 ×Holotrichs^1^	0.001
*t*11-18:1	-3.40±1.371 + 1.25±0.0243 × Total ciliates^1^	<0.001
	-2.73±1.514 + 1.17±0.271 × Entodiniomorphids^1^	<0.001
	-3.73±1.463 + 1.37±0.261 × *Entodinium*^1^	<0.001
*t*10/*t*11 ratio	1.55±0.673–0.113±0.0526 × Total ciliates^1^	0.036
*c*9,*t*11-18:2	-0.816±0.4438 +0.355±0.0763× Total ciliates^1^	<0.001
	-0.794±0.4168 + 0.354±0.0716× Entodiniomorphids^1^	<0.001
	-0.850±0.4023 + 0.371±0.0702× *Entodinium*^1^	<0.001
SFA	42.6±0.62–0.134±0.0660 × Holotrichs^1^	0.046
	42.6±0.52–0.131±0.0669 × *Isotricha*^1^	0.054
n-3 PUFA	1.37±0.133–0.094±0.0231× Total ciliates^1^	<0.001
	0.891±0.1032–0.003±0.0007 ×Holotrichs^1^	<0.001
	1.11±0.099–0.042±0.0099 × Entodiniomorphids^1^	<0.001
	1.16±0.081–0.057±0.0138× *Entodinium*^1^	<0.001
n-6 PUFA	9.07±0.278–0.140±0.0487 ×Holotrichs^1^	0.005
	12.1±1.26–0.597±0.2218 × Entodiniomorphids^1^	0.009
	11.5±1.17–0.500±0.2079× *Entodinium*^1^	0.019
n-3 LC-PUFA	0.598±0.0910–0.038±0.0163 ×Total ciliates^1^	0.024
	0.614±0.0994–0.041±0.0177 × Entodiniomorphids^1^	0.024
	0.583±0.1012–0.036±0.0182× *Entodinium*^1^	0.053
PUFA	11.8±0.86–0.397±0.1704× Total ciliates^1^	0.033
	14.1±1.21–0.782±0.2158 × Entodiniomorphids^1^	<0.001
	11.5±0.74–0.348±0.1470× *Entodinium*^1^	0.021

SFA, Saturated fatty acids; PUFA, Polyunsaturated fatty acids;n-6 PUFA, 18:2 n-6 + 20:4 n-6 + 22:4 n-6; n-3 PUFA,18:3 n-3 + 20:5 n-3 + 22:5 n-3 + 22:6 n-3; LC n-3 PUFA, 20:5 n-3 + 22:5 n-3 + 22:6 n-3.

^1^ log_10_ cells/ml

In meat (Table 6), the *c*9-18:1(*P* = 0.040), the 18:2 n-6 (*P* = 0.001), the *t*10/*t*11 ratio (*P* = 0.036), the n-3 PUFA (*P* <0.001), the n-3 LC-PUFA (*P* = 0.024) and the PUFA (*P* = 0.033) decreased linearly with the concentration of Total ciliates. Oleic acid (*c*9-18:1) (*P* = 0.035), 18:3 n-3 (*P* = 0.001), SFA (*P* = 0.046), n-3 PUFA (*P* < 0.001) and n-6 PUFA (*P* = 0.005) decreased linearly with Holotrichs. Stearic acid (*P* = 0.005), *c*9-18:1(*P* = 0.009), 18:2 n-6 (*P* < 0.001), n-3 PUFA (*P* <0.001), n-6 PUFA (*P* = 0.009), n-3 LC-PUFA (*P* = 0.024) and PUFA (*P* < 0.001) decreased linearly with Entodiniomorphids. At the genus level, it were also observed linear decreases of *c*9-18:1(*P* = 0.014) and SFA (*P* = 0.054) with *Isotricha*, of *c*9-18:1 (*P* = 0.011), 18:2 n-6 (*P* = 0.003), n-3 PUFA (*P* <0.001), n-6 PUFA (*P* = 0.019), n-3 LC-PUFA (*P* = 0.054) and PUFA (*P* = 0.021) with *Entodinium* and of *c*9-18:1(*P* < 0.001) with *Epidinium*. Vaccenic acid (*t*11-18:1) (*P* <0.001) and of the *c*9,*t*11-18:2 (*P* <0.001) increased linearly with Total ciliates, Entodiniomorphids and *Entodinium*.

### Effect of the type of protozoa population on rumen and meat FA

The results of the effect of the type of the protozoa population on the FA content of rumen are presented in Table 7. The type of population influenced the individual proportion of 18:0 (*P* = 0.003), *c*9-18:1 (*P* = 0.004), 18:3 n-3 (*P*< 0.001), *t*10-18:1 (*P* = 0.024) and the sum of SFA (*P* = 0.010). The A type population resulted in the lowest value for 18:0, low proportions of *t*10-18:1 and SFA and in the highest value for 18:3 n-3 proportion and high *c*9-18:1. The B type population resulted in the lowest proportion of 18:3 n-3, intermediate proportions of *c*9-18:1, *t*10-18:1 and SFA and high proportion of 18:0. The O type population resulted in the low proportion of *c*9-18:1, intermediate proportion of 18:3 n-3 and high value for 18:0, *t*10-18:1 and SFA proportions.

**Table 7:** Effect of the type of protozoa population in rumen fatty acids.

	Protozoa population type			*P*-value
	A	B	O	
**Rumen FA(g/100gFA)**				
18:0	27.4±2.18a	35.6±2.21b	37.1±1.78b	0.003
*c*9-18:1	11.4±0.43b	10.3±0.10ab	9.14±0.494a	0.004
18:2 n-6	7.04±1.033	4.63±0.532	4.56±0.502	0.090
18:3 n-3	1.54±0.142c	0.58±0.056a	0.84±0.082b	<0.001
*t*10-18:1	1.04±0.227a	1.91±0.432ab	2.29±0.443b	0.024
*t*11-18:1	12.9±1.65	11.6±1.33	11.6±1.03	0.776
*t*10/*t*11 ratio	0.13±0.039	0.17±0.066	0.21±0.061	0.548
c9,t11-18:2	0.35±0.052	0.32±0.046	0.38±0.042	0.604
*t*10,*c*12-18:2	0.073±0.0190	0.032±0.0226	0.053±0.0140	0.375
18:1 BI	21.5±1.36	22.7±1.05	21.3±1.06	0.596
18:2 BI	1.15±0.118	1.07±0.064	1.35±0.123	0.139
SFA	50.3±0.56a	54.0±2.07ab	55.0±1.66b	0.010
PUFA	7.58±1.148	5.17±0.579	5.23±0.547	0.154
**Total FA (mg/g DM)**	85.0±2.44	74.9±3.77	81.6±1.77	0.084
**RBH completeness**	64.2±1.54	63.2±2.52	65.0±2.11	0.860

SFA, Saturated fatty acids; PUFA, Polyunsaturated fatty acids; n-6 PUFA, 18:2 n-6 + 20:4 n-6 + 22:4 n-6; n-3 PUFA,18:3 n-3 + 20:5 n-3 + 22:5 n-3 + 22:6 n-3; LC n-3 PUFA, 20:5 n-3 + 22:5 n-3 + 22:6 n-3.

Table 8 shows the results of the effect of the type of ciliates population on the FA of Lt muscle. The type of population influenced the individual proportions of 18:3 n-3 (*P* = 0.009), *t*10-18:1 (*P* = 0.009), the *t*10/*t*11 ratio (*P* = 0.006) and the sums of SFA (*P*< 0.001) and of n-3 PUFA (*P* = 0.011). The A type population resulted in the lowest value for *t*10-18:1 and low values of *t*10/*t*11 ratio and n-3 PUFA, in the highest SFA and in a high proportion of 18:3 n-3. The B type population, resulted in the lowest proportion of 18:3 n-3, low SFA and n-3 PUFA proportions, intermediate t10/t11 ratio and a high proportion of *t*10-18:1. The O type population resulted in low proportion of SFA, in the highest value for n-3 PUFA and in high 18:3 n-3, *t*10-18:1 and *t*10/*t*11 ratio in muscle.

**Table 8:** Effect of the type of protozoa population in meat fatty acids.

	Protozoa population type			*P*-value
	A	B	O	
**Muscle FA (g/100g FA)**				
18:0	15.9±0.30	16.1±0.14	16.0±0.24	0.729
*c*9-18:1	32.1±0.76	31.1±0.34	31.2±0.36	0.486
18:2 n-6	6.54±0.476	7.17±0.266	7.22±0.251	0.440
18:3 n-3	0.56±0.025b	0.45±0.033a	0.57±0.025b	0.009
*t*10-18:1	1.38±0.230a	2.30±0.290b	2.35±0.248b	0.009
*t*11-18:1	4.64±0.540	4.05±0.409	3.94±0.252	0.505
*t*10/*t*11 ratio	0.29±0.060a	0.53±0.098ab	0.68±0.175b	0.006
*c*9,*t*11-18:2	1.45±0.123	1.18±0.114	1.21±0.062	0.188
*t*10,*c*12-18:2	0.005±0.0076	0.021±0.0061	0.010±0.0048	0.219
SFA	43.2±0.06b	42.6±0.29a	42.0±0.29a	<0.001
*cis*-MUFA	35.5±0.77	34.6±0.43	34.6±0.39	0.592
n-6 PUFA	8.30±0.494	9.03±0.330	9.01±0.286	0.413
n-3 PUFA	0.74±0.027a	0.74±0.028a	0.84±0.028b	0.011
n-6 LC-PUFA	1.70±0.091	1.68±0.106	1.60±0.080	0.688
n-3 LC-PUFA	0.41±0.035	0.36±0.018	0.42±0.019	0.089
PUFA	9.29±0.550	10.1±0.36	10.1±0.32	0.401
**Total FA (mg/g DM)**	89.9±5.05	98.9±4.98	94.6±3.23	0.455

SFA, Saturated fatty acids; PUFA, Polyunsaturated fatty acids; n-6 PUFA, 18:2 n-6 + 20:4 n-6 + 22:4 n-6; n-3 PUFA,18:3 n-3 + 20:5 n-3 + 22:5 n-3 + 22:6 n-3; LC n-3 PUFA, 20:5 n-3 + 22:5 n-3 + 22:6 n-3.

## Discussion

Rumen ciliate protozoa are usually present on rumen at numbers ranging between 10^4^ to 10^6^ cells/ml [14] but host diet strongly affects their numbers which can range from zero (defaunation) up to 5 × 10^6^ cells/ ml [31]. Dietary lipids rich in PUFA or in lauric acid, excerpt toxic effects on rumen protozoa, and may be used as supplement in ruminant diets to reduce methane emissions [7, 8, 32, 33] or as promoters of *t*11-18:1 and *c*9,*t*11-18:2 in ruminant products [1]. All the diets used in the three experiments were supplemented with 6% of soybean oil. Despite this, 88.4% of the lambs were faunated, with protozoa numbers ranging between 9.0 × 10^3^cells/ ml and 1.2 × 10^7^cells/ml, which suggest that in some samples the concentration of protozoa was even above the upper value referred by [14].

The abundance of ciliate protozoa is highly influenced by rumen pH [34, 35] and values below 5.5 above 15 h/day generally cause the elimination of protozoa [34]. In the present study, we have no data regarding the daily fluctuations of rumen pH. The rumen pH, was determined only once, immediately after the slaughter and the lambs were not submitted to pre-slaughter fasting. The values ranged between 5.25 and 7.67and correlated positively with rumen ciliates, which is in line with the literature [34, 35].

Usually 5 to 6 genera of rumen ciliates are observed in each faunated rumen [36] but in this study most cases (n = 67) only presented 1 to 3 ciliate genera in rumen. Host diet plays a crucial role not only in the abundance but also in the community structure of rumen protozoa [31, 37, 38]. The ciliate genera composition observed probably reflects the balance between starch, soluble sugars and structural carbohydrates available in the rumen. Our data also show a strong variability among individuals, which is in accordance to [39] and [38]. A high variability among lambs was observed for both the concentration of ciliates and for the genera composition and frequently, animals fed the same diet presented different ciliate densities and community structures.

The relationships between rumen protozoa and lipid metabolism in rumen are still not fully clarified [8]. However, independently of the mechanisms involved, our results suggest that rumen ciliates may modulate the RBH, by favouring its first steps and reducing the last step, which results in the accumulation 18:1-BI, as it was suggested by[18]. In fact, the present results show negative relationships between the concentration of ciliates and the proportions of PUFA, 18:2-BI, 18:0 and RBH completeness in rumen and positive relationships between *Isotricha*, *Entodinium* and *Epidinium* genera and 18:1-BI in rumen. Moreover, also the *c*9-18:1 in muscle decreased linearly with the protozoa concentration, independently of the ciliates genera. In lipid supplemented ruminants, the *c*9-18:1 is derived mostly from endogenous desaturation of rumen derived 18:0 [1], thus the negative relationship of muscle *c*9-18:1 with protozoa is consistent with a reduced rumen outflow of 18:0.

Rumen concentration and muscle deposition of *t*11-18:1 and *c*9,*t*11-18:2 increased linearly. These results confirm the previous observations that faunated lambs tended to have higher proportion of *t*11-18:1 in the muscle when compared to the defaunated lambs [18]. Rumen protozoa accumulate high levels of the *t*11-18:1 and *c*9,*t*11-18:2 in their membranes [19, 20, 40, 41] and its association with *t*11-18:1 and *c*9,*t*11-18:2 in meat and milk was hypothesized [18, 40, 42]. Our results are in line with that hypothesis, showing a clear positive association between ciliates concentration in rumen and the deposition of *t*11-18:1 and *c*9,*t*11-18:2 in meat.

The incorporation into the membranes of the ciliate protozoa of both *t*11-18:1 and *c*9, *t*11-18:2, allows them to escape to the subsequent hydrogenation, increasing the amount of those AF flowing from the rumen, becoming available for absorption and deposition in the tissues animals. The amount and rate of biomass protozoa flow from rumen is being under intense debate [8], due to the selective retention of these microorganisms within the rumen[42–44]. It seems that the retention of ciliates in rumen is not similar for every protozoa group[8]. While Holotrichs have a strong chemotaxis toward sugars and after a meal migrate to the ventral reticulo-rumen, avoiding to be washed out, Entodiniomorphids have a moderate chemotaxis toward glucose and peptides and are uniformly distributed in ruminal content do not show the same affinity to the rumen wall as Holotrichs, and thus flow out of the rumen with the particulate matter [8, 45, 46]. The results of the present study are compatible with that dynamics of protozoa in rumen. The strong positive relationships between the Entodiniomorphids, namely the genus *Entodinium*, and *t*11-18:1 and *c*9,*t*11-18:2 in meat suggest a high availability of *t*11-18:1 for duodenal absorption and deposition in tissues. The positive relationships that were observed between Holotrichs and *t*11-18:1 and *c*9,*t*11-18:2 in rumen, were not maintained for meat.

Another mechanism that may contribute to explain the relationships among ciliates and *t*11-18:1 and *c*9,*t*11-18:2 is their effect as stabilizing agents of rumen fermentation of non-structural carbohydrates, contributing to the maintenance of rumen pH values above 6.0, which are compatible to the growth and activity of the cellulolytic biohydrogenating bacteria [2]. Holotrichs metabolize preferentially soluble readily available carbohydrates [47], which absorb and accumulate within the cell, preventing its rapid fermentation by bacteria [9, 47]. Our results suggest that genus *Isotricha* may be particularly relevant in this context because it was the only for whom linear regression was significant. Moreover, Entodiniomorphids, particularly from *Entodinium* genus, engulf and metabolize particulate matter, and are responsible by the removal of large amounts of starch and feed particles from rumen liquor [9]. Due to their fast up take of starch granules, they reduce the availability of substrate to the amilolytic bacteria, allowing a better control of starch fermentation and reducing rumen pH fluctuations [9].

As far as we know, this is the first study reporting the relationships between rumen protozoa and the occurrence of the *trans*-10 shifted RBH pathway evaluated by the *t*10-18:1 and *t*10/t11 ratio in rumen content and in meat. The proportion of *t*10-18:1 and the *t*10/*t*11 ratio showed negative and significant correlations with total ciliates and total Entodinomorphids and the regressions analysis reinforced these results. Considering individual genera, the correlations with *t*10-18:1 and with the *t*10/*t*11 ratio were also generally negative. However, when linear regression analysis was applied to rumen data, only for *Entodinium* and *Epidinium* the regression coefficients were significant. These results suggest that, in the set of genera considered, these two Entodinomorphids are the most associated to the reduction of the *trans*-10 shifted pathway in the rumen.

In addition, the ANOVA results suggest that the presence of the protozoa type A population is related to a low *t*10-18:1 content and to a *t*10/*t*11 ratio in the rumen and in meat. The Type A protozoa population is characterized by the presence of the genus *Polyplastron*, which has a large cell size and a high predatory and digestive activities for most carbohydrates [48]. These characteristics may contribute to impair rumen the conditions that favour the *t*10 shifted pathway.

This study was performed with data from three previous experiments of our group and was not specifically designed to evaluate the effects of protozoa in lipid rumen metabolism. However, our results suggest a close relationship between the concentration of ciliate protozoa in rumen and the accumulation of 18:1 BI in rumen and meat, namely *t*11-18:1 and reducing the probability and intensity of the *trans*-10 shift in rumen, what positively impacts the nutritional value of ruminant´s meat. The effects of diet composition on rumen ciliates concentration and community structure should be considered when nutritional strategies are planned to improve the nutritional value of ruminant products. More research is needed to clarify and to explore the relationships observed between ciliate community and RBH pathways and completeness, including other genera that were not explored in this study.

## Conclusions

The relationships between protozoa community and lipid rumen metabolism was associated with the type of ciliates and Entodinomorphids were more linked with RBH than Holotrichs.

Rumen protozoa were positively linked with *t*11-18:1 and *c*9,*t*11-18:2 and negatively related with the *t*10-18:1, with *trans*-10 shift, with RBH completeness and with 18:0.

The concentration of ciliate protozoa in rumen was positively related to the *t*11-biohydrogenation pathway and to a healthier fatty acid profile in lamb meat.

## Supporting information

S1 Table Ingredients, chemical composition and fatty acid (FA) profile of the experimental diets from the Experiment 1. (PDF)

S2 Table Ingredients, chemical composition and fatty acid (FA) profile of the experimental diets from the Experiment 2. AP0, alfalfa pellets and 0% barley grain; AP35, alfalfa and 35% of barley grain; AP65, alfalfa pellets and 65% of barley grain; AP100, alfalfa pellets and 100% of barley grain; AH0, alfalfa hay and 0% of barley grain; AH35, alfalfa hay and 35% of barley grain; AH65, alfalfa hay and 65% of barley grain; AH100, alfalfa hay and 100% of barley grain.
(PDF)

S3 Table Ingredients, chemical composition and fatty acid (FA) profile of the experimental diets from the Experiment 3. A0.5, alfalfa as forage source and 0.5% of SB as buffer; A2.0, alfalfa as forage source and 2.0% of SB as buffer; ALV, alfalfa as forage source and 0.5% of SB plus 1.5% Levucell^®^ SC20 as buffer; R0.5 –ryegrass as forage source and 0.5% of SB as buffer; R2.0, ryegrass as forage source and 2.0% of SB as buffer.
(PDF)

## References

[1] Bessa RJB, Alves SP, Santos-Silva J. Constraints and potentials for the nutritional modulation of the fatty acid composition of ruminant meat. Europ J Lipid Sci Technol. 2015;117:1325–44. 10.1002/ejlt.201400468

[2] Jenkins TC, Wallace RJ, Moate PJ, Mosley EE. Board-invited review: Recent advances in biohydrogenation of unsaturated fatty acids within the rumen microbial ecosystem. J Anim Sci. 2008;86(2):397–412. Epub 2007/11/29. 10.2527/jas.2007-0588 . 18042812

[3] Martin SA, Jenkins TC. Factors affecting conjugated linoleic acid and trans-C18:1 fatty acid production by mixed ruminal bacteria. J Anim Sci. 2002 80(12):3347–52. 10.2527/2002.80123347x 12542176

[4] Griinari JM, Bauman DE. Biosynthesis of conjugated linoleic acid and its incorporation into meat and milk in ruminants In: Yuraweczmp MP, Mossoba MM, Kramer JKG, Pariza MW, Nelson G, editors. Advances in conjugated linileic acid research. Champagin, IL: American Oil Chemists Society Press; 1999 p. 180–200.

[5] Aldai N, Renobales M, Barron LJR, Kramer JKG. What are the trans fatty acids issues in foods after discontinuation of industrially produced trans fats? Ruminant products, vegetable oils, and synthetic supplements. Europ J Lipid Sci Technol. 2013;115:1378–401. 10.1002/ejlt.201300072

[6] Shingfield KJ, Griinari JM. Role of biohydrogenation intermediates in milk fat depression. Europ J Lipid Sci Technol. 2007;109(8):799–816. 10.1002/ejlt.200700026

[7] Hook SE, Wright A-DG, McBride BW. Methanogens: Methane Producers of the Rumen and Mitigation Strategies. Archaea. 2010;2010:11 10.1155/2010/945785 21253540 PMC3021854

[8] Newbold CJ, de la Fuente G, Belanche A, Ramos-Morales E, McEwan NR. The Role of Ciliate Protozoa in the Rumen. Frontiers in Microbiology. 2015;6:1313. 10.3389/fmicb.2015.01313 PMC4659874 26635774

[9] Williams AG, Coleman GS. The rumen protozoa In: Hobsen PN, Stewart CS, editors. The rumen microbial microsystem. Dorbrecht, Netherlands: Springer; 1997.

[10] Nagaraja TG, Titgemeyer EC. Ruminal Acidosis in Beef Cattle: The Current Microbiological and Nutritional Outlook. J Dairy Sci. 2007;90:E17–E38. 10.3168/jds.2006-478. 17517750

[11] Noziere P, Ortigues-Marty I, Loncke C, Sauvant D. Carbohydrate quantitative digestion and absorption in ruminants: from feed starch and fibre to nutrients available for tissues. Animal. 2010;4(7):1057–74. Epub 2010/07/01. 10.1017/S1751731110000844 . 22444609

[12] Newbold CJ, Williams AG, Chamberlain DG. The in vitro metabolism of d,l-lactic acid by rumen microorganisms. Journal of Science and Food Agriculture. 1987;38(1):9–18.

[13] Harfoot CG, Hazelwood GP. Lipid metabolism in the rumen In: Hobson PN, editor. The rumen microbial ecosystem. London, UK: Elsevier Science Publishing; 1997 p. 382–426.

[14] Nagaraja TG. Microbiology of the rumen In: Millen D, De Beni Arrigoni M, Lauritano Pacheco RD, editors. Ruminology. Switzerland: Springer International Publish; 2016 p. 39–61.

[15] Huws SA, Kim EJ, Kingston-Smith AH, Lee MR, Muetzel SM, Cookson AR, et al Rumen protozoa are rich in polyunsaturated fatty acids due to the ingestion of chloroplasts. FEMS microbiology ecology. 2009;69(3):461–71. Epub 2009/07/09. 10.1111/j.1574-6941.2009.00717.x . 19583786

[16] Boeckaert C, Morgavi DP, Jouany JP, Maignien L, Boon N, Fievez V. Role of the protozoan Isotricha prostoma, liquid-, and solid-associated bacteria in rumen biohydrogenation of linoleic acid. Animal. 2009;3(7):961–71. Epub 2009/07/01. 10.1017/S1751731109004285 . 22444816

[17] Kim EJ, Huws SA, Lee MRF, Scollan ND. Dietary Transformation of Lipid in the Rumen Microbial Ecosystem. Asian-Australasian Journal of Animal Sciences. 2009;22(9):1341–50. 10.5713/ajas.2009.r.11

[18] Yanez-Ruiz DR, Williams S, Newbold CJ. The effect of absence of protozoa on rumen biohydrogenation and the fatty acid composition of lamb muscle. British Journal of Nutrition. 2007;97(5):938–48. Epub 2007/03/27. 10.1017/S0007114507675187 . 17381978

[19] Devillard E, McIntosh FM, Newbold CJ, Wallace RJ. Rumen ciliate protozoa contain high concentrations of conjugated linoleic acids and vaccenic acid, yet do not hydrogenate linoleic acid or desaturate stearic acid. British Journal of Nutrition. 2007;96(4):697–704. Epub 03/08. 17010229

[20] Sultana H, Miyazawa K, Kanda S, Itabashi H. Fatty acid composition of ruminal bacteria and protozoa, and effect of defaunation on fatty acid profile in the rumen with special reference to conjugated linoleic acid in cattle. Animal Science Journal. 2011;82(3):434–40. Epub 2011/05/28. 10.1111/j.1740-0929.2010.00854.x . 21615837

[21] Santos-Silva J, Francisco A, Alves SP, Portugal APV, Dentinho MT, Almeida AM, et al Effect of dietary neutral detergent fiber source on lambs growth, meat quality and biohydrogenation intermediates. Meat Sci. 2019;147:28–36. 10.1016/j.meatsci.2018.08.015 30196198

[22] Francisco A, Alves SP, Portugal PV, Pires VMR, Dentinho MT, Alfaia C, et al Effect of feeding lambs with a tanniferous shrub (rockrose) and a vegetable oil blend on fatty acid composition of meat lipids. Animal. 2016:1–13. 10.1017/S1751731116001129 27306827

[23] Dehority BA. Evaluation of subsampling and fixation procedures used for counting rumen protozoa. Applied and Environment Microbiology. 1984;48(1):182–5. 10.1128/aem.48.1.182-185.1984 PMC240360 6476828

[24] Hogimoto K, Imai S. Atlas of rumen microbiology. Tokyo: Japan Scientific Societies Press; 1981.

[25] Alves SP, Santos-Silva J, Cabrita ARJ, Fonseca AJM, Bessa RJB. Detailed Dimethylacetal and Fatty Acid Composition of Rumen Content from Lambs Fed Lucerne or Concentrate Supplemented with Soybean Oil. PLOS ONE. 2013;8(3):e58386 10.1371/journal.pone.0058386 23484024 PMC3587585

[26] Jerónimo E, Alves SP, Prates JAM, Santos-Silva J, Bessa RJB. Effect of dietary replacement of sunflower oil with linseed oil on intramuscular fatty acids of lamb meat. Meat Sci. 2009;83:499–505. 10.1016/j.meatsci.2009.06.033 . 20416670

[27] Alves SP, Raundrup K, Cabo Â, Bessa RJB, Almeida AM. Fatty Acid Composition of Muscle, Adipose Tissue and Liver from Muskoxen (Ovibos moschatus) Living in West Greenland. PLOS ONE. 2015;10(12):e0145241 10.1371/journal.pone.0145241 26678792 PMC4683068

[28] Alves SP, Bessa RJB. Comparison of two gas-liquid chromatograph columns for the analysis of fatty acids in ruminant meat. Journal of Chromatography A,. 2009;1216::5130–9. 10.1016/j.chroma.2009.04.079 19446820

[29] Vahmani P, Rolland DC, Gzy IKE, Dugan MER. Non-conjugated cis/trans 18:2 in Beef Fat are Mainly Δ-9 Desaturation Products of trans-18:1 Isomers. Lipids,. 2016;51:1427–33 10.1007/s11745-016-4207-0 27853932

[30] Alves SP, Francisco A, Costa M, Santos-Silva J, Bessa RJB. Biohydrogenation patterns in digestive contents and plasma of lambs fed increasing levels of a tanniferous bush (Cistus ladanifer L.) and vegetable oils. Anim Feed Sci Technol. 2017;225:157–72. 10.1016/j.anifeedsci.2017.01.018

[31] Church DC. Digestive Physiology and Nutrition of Ruminants: Nutrition: O & B Books; 1979 452 p.

[32] Guyader J, Eugene M, Noziere P, Morgavi DP, Doreau M, Martin C. Influence of rumen protozoa on methane emission in ruminants: a meta-analysis approach. Animal. 2014;8(11):1816–25. Epub 2014/07/31. 10.1017/S1751731114001852 . 25075950

[33] Toprak NN. Due fats reduce methane emission by ruminants. Animal Science Papers and Reports. 2015;33(4):305–21.

[34] Franzolin R, Dehority BA. Effects of ruminal PH and feed intake on defaunation in sheep fed high concentrate diets. Rev Bras Zootecn. 1996;25(6):1207–15.

[35] Dehority BA. Effect of pH on viability of Entodinium caudatum, Entodinium exiguum, Epidinium caudatum, and Ophryoscolex purkynjei in vitro. Journal of Eukaryotic Microbiology. 2005;52(4):339–42. Epub 2005/07/15. 10.1111/j.1550-7408.2005.00041.x . 16014011

[36] Bohatier J. The rumen protozoa: taxonomy, cytology and feeding behaviour In: Jouany J, editor. Rumen microbial metabolism and ruminant digestion. Paris, France: INRA; 1991 p. 27–38.

[37] Coleman GS. The role of rumen protozoa in the metabolism of ruminants given tropical feeds. Tropical Animal Production. 1979;4(3):199–213.

[38] Henderson G, Cox F, Ganesh S, Jonker A, Young W. Rumen microbial community composition varies with diet and host, but a core microbiome is found across a wide geographical range. Scientific Reports [Internet]. 2015; 5:[14567 p.]. 10.1038/srep14567 26449758 PMC4598811

[39] Weimer PJ. Redundancy, resilience, and host specificity of the ruminal microbiota: implications for engineering improved ruminal fermentations. Frontiers of Microbiology. 2015;6:296 Epub 2015/04/29. 10.3389/fmicb.2015.00296 . 25914693 PMC4392294

[40] Or-Rashid MM, Odongo NE, McBride BW. Fatty acid composition of ruminal bacteria and protozoa, with emphasis on conjugated linoleic acid, vaccenic acid, and odd-chain and branched-chain fatty acids. J Anim Sci. 2007;85(5):1228–34. Epub 2006/12/06. 10.2527/jas.2006-385 . 17145972

[41] Cièslak A, Miltko R, Belzecki G, Szumacher-Strabel M, Michalowski T. Rumen Ciliates Entodinium caudatum, Eudiplodinium maggii and Diploplastron affine: a Potential Reservoir of Unsaturated Fatty Acids for the Host. Acta Protozoologica 2009;48 (4):335–40.

[42] Huws SA, Lee MR, Kingston-Smith AH, Kim EJ, Scott MB, Tweed JK, et al Ruminal protozoal contribution to the duodenal flow of fatty acids following feeding of steers on forages differing in chloroplast content. British Journal of Nutrition. 2012;108(12):2207–14. Epub 2012/03/02. 10.1017/S0007114512000335 . 22377337

[43] Dehority BA, Tirabasso PA. Factors Affecting the Migration and Sequestration of Rumen Protozoa in the Family Isotrichidae Journal of General Microbiology. 1989;135:539–48.

[44] Hook SE, Dijkstra J, Wright AD, McBride BW, France J. Modeling the distribution of ciliate protozoa in the reticulo-rumen using linear programming. J Dairy Sci. 2012;95(1):255–65. Epub 2011/12/24. 10.3168/jds.2011-4352 . 22192205

[45] Diaz HL, Karnati SKR, Lyons MA, Dehority BA, Firkins JL. Chemotaxis toward carbohydrates and peptides by mixed ruminal protozoa when fed, fasted, or incubated with polyunsaturated fatty acids. J Dairy Sci. 2014;97(4):2231–43. 10.3168/jds.2013-7428. 24534499

[46] Hook SE, France J, Dijkstra J. Further assessment of the protozoal contribution to the nutrition of the ruminant animal. Journal Theoretical Biology. 2017;416:8–15. Epub 2016/12/23. 10.1016/j.jtbi.2016.12.007 . 28007554

[47] Williams AG. Rumen holotrich ciliate protozoa. Microbiological Reviews. 1986;50(1):25–49. 3083220 10.1128/mr.50.1.25-49.1986 PMC373052

[48] Jouany JP, Zainab B, Senaud J, Groliere CA, Grain J, Thivend P. Rôle of the rumen ciliate protozoa Polyplastron multivesiculatum, Entodinium sp. and Isotricha prostoma in the digestion of a mixed diet in sheep. Reprod Nutr Dev. 21(6A):871–84. 6818640 10.1051/rnd:19810701

