# Supplementary material for: Relationship between rumen ciliate protozoa and biohydrogenation fatty acid profile in rumen and meat of lambs
Source: PLoS One. 2019 Sep 6;14(9):e0221996. doi: 10.1371/journal.pone.0221996 (PMC6730912; doi:10.1371/journal.pone.0221996)
Supplement: S1 Table — (PDF) [file pone.0221996.s001.pdf]

	Diets		
	**20% Alfalfa**	**40% Alfalfa**	**60% Alfalfa**
**Ingredients, g/kg**			
Alfalfa pellets	200	400	600
Soy hulls	338	235	90
Citrus pulp	70	56	30
Beet pulp	120	54	40
Soybean meal	187	170	155
Soybean oil	60	60	60
Calcium carbonate	13	13	13
Sodium bicarbonate	5	5	5
Salt	4	4	4
Premix	3	3	3
**Chemical composition, g/kg DM**			
DM	907	914	917
CP	177	169	183
Ether extract	79	76	71
Starch	56	55	58
Sugar	95	102	108
NDF	437	432	418
**FA profile, g/100g FA**			
16:0	12.7	12.4	12.4
18:0	4.1	4.1	4.1
*c*9-18:1	22.9	22.5	22.5
*c*11-18:1	2.0	2.0	2.0
18:2n-6	51.3	51.7	50.5
18:3n-3	5.9	6.5	5.9

DM, dry matter; FA, fatty acids
