# Supplementary material for: Relationship between rumen ciliate protozoa and biohydrogenation fatty acid profile in rumen and meat of lambs
Source: PLoS One. 2019 Sep 6;14(9):e0221996. doi: 10.1371/journal.pone.0221996 (PMC6730912; doi:10.1371/journal.pone.0221996)
Supplement: S2 Table — AP0, alfalfa pellets and 0% barley grain; AP35, alfalfa and 35% of barley grain; AP65, alfalfa pellets and 65% of barley grain; AP100, alfalfa pellets and 100% of barley grain; AH0, alfalfa hay and 0% of barley grain; AH35, alfalfa hay and 35% of barley grain; AH65, alfalfa hay and 65% of barley grain; AH100, alfalfa hay and 100% of barley grain. (PDF) [file pone.0221996.s002.pdf]

	Diets							
	AP0	AP35	AP65	AP100	AH0	AH35	AH65	AH100
**Ingredients, g/kg**								
Alfalfa pellets	400	400	400	400	-	-	-	-
Alfalfa hay	-	-	-	-	400	400	400	400
Barley grain	0	112	213	330	0	112	213	330
Soy hulls	106	71	38	0	106	71	38	0
Citrus pulp	107	71	38	0	107	71	38	0
Beet pulp	107	71	38	0	107	71	38	0
Soybean meal	180	175	173	170	180	175	172	170
Soybean oil	60	60	60	60	60	60	60	60
Calcium carbonate	13	13	13	13	13	13	13	13
Sodium bicarbonate	20	20	20	20	20	20	20	20
Salt	4	4	4	4	7	7	7	7
Premix	3	3	3	3	3	3	3	3
**Chemical composition, g/kg DM**								
DM	897	902	902	899	900	898	900	900
CP	197	194	191	195	184	185	193	172
Ether extract	80	80	83	75	74	74	73	72
Starch	31	102	158	227	29	96	156	209
Sugar	66	65	54	52	85	74	67	56
NDF	341	329	317	272	343	318	280	265
**FA profile, g/100g FA**								
16:0	14.6	15.2	14.9	14.7	17.1	16.0	15.8	16.0
18:0	3.4	3.1	3.0	3.1	3.3	3.2	3.1	2.9
*c*9-18:1	24.5	26.0	25.6	25.3	24.8	24.0	24.0	25.0
*c*11-18:1	1.8	1.9	1.8	1.5	2.1	2.0	1.9	2.1
18:2n-6	49.0	47.9	48.7	49.2	46.0	46.0	47.5	47.0
18:3n-3	4.5	3.6	3.8	3.8	4.3	3.9	4.3	3.9

DM, dry matter; FA, fatty acids
