# Supplementary material for: Relationship between rumen ciliate protozoa and biohydrogenation fatty acid profile in rumen and meat of lambs
Source: PLoS One. 2019 Sep 6;14(9):e0221996. doi: 10.1371/journal.pone.0221996 (PMC6730912; doi:10.1371/journal.pone.0221996)
Supplement: S3 Table — A0.5, alfalfa as forage source and 0.5% of SB as buffer; A2.0, alfalfa as forage source and 2.0% of SB as buffer; ALV, alfalfa as forage source and 0.5% of SB plus 1.5% Levucell® SC20 as buffer; R0.5 –ryegrass as forage source and 0.5% of SB as buffer; R2.0, ryegrass as forage source and 2.0% of SB as buffer. (PDF) [file pone.0221996.s003.pdf]

	Diets				
	**A0.5**	**A2.0**	**ALV**	**R0.5**	**R2.0**
**Ingredients, g/kg**					
Alfalfa pellets	400	400	400	-	-
Ryegrass hay	-	-	-	400	400
Barley grain	210	197.5	112	203.5	192.5
Soy hulls	45	42.5	71	45	42
Citrus pulp	45	42.5	71	45	42
Beet pulp	45	42.5	71	45	42
Soybean meal	120	125	175	170	175
Wheat bran	50	50	15	-	-
Soybean oil	60	60	60	60	60
Calcium carbonate	13	13	13	13	13
Sodium bicarbonate	5	20	5	5	20
Salt	4	4	4	4	4
Premix	3	3	3	3	3
**Chemical composition, g/kg DM**					
DM	894	890	902	894	889
CP	178	181	194	185	174
Ether extract	86	76	80	83	85
Starch	175	187	102	172	162
Sugar	73	84	65	113	103
NDF	355	342	329	354	373
**FA profile, g/100g FA**					
16:0	14.4	15.0	15.2	14.3	14.8
18:0	4.2	4.9	3.1	4.5	4.2
*c*9-18:1	22.8	22.5	26.0	23.4	24.0
*c*11-18:1	1.7	1.7	1.9	1.8	1.8
18:2n-6	51.2	49.6	47.9	50.3	49.9
18:3n-3	5.6	5.7	3.6	5.7	5.7

DM, dry matter; FA, fatty acids
